# miR-744 enhances type I interferon signaling pathway by targeting PTP1B in primary human renal mesangial cells

**DOI:** 10.1038/srep12987

**Published:** 2015-08-11

**Authors:** Xiaoyan Zhang, Xiao Han, Yuanjia Tang, Yanfang Wu, Bo Qu, Nan Shen

**Affiliations:** 1 Shanghai Institute of Rheumatology, Department of Rheumatology, Ren Ji Hospital, School of Medicine, Shanghai Jiao Tong University, Shanghai, China; 2 Institute of Health Sciences, Shanghai Institutes for Biological Sciences (SIBS) & Shanghai Jiao Tong University School of Medicine (SJTUSM), Chinese Academy of Sciences (CAS), Shanghai, China; 3 Division of Rheumatology and the Center for Autoimmune Genomics and Etiology (CAGE),Cincinnati Children’s Hospital Medical Center, Cincinnati, Ohio, United States of America.

## Abstract

Renal mesangial cells (RMCs) constitute a population of cells in glomerular mesangium. Inflammatory cytokines produced by RMCs play a vital role in renal inflammation. miRNAs are key regulators of inflammatory cytokine expression. The abnormal expression of renal miRNAs and the consequent changes in inflammatory signal transduction are closely associated with renal inflammation. However, our knowledge of the functions of renal miRNAs is still limited. In this study, we investigated the role of miR-744 in type I interferon (IFN) signaling pathway in primary human RMCs. We show that overexpression of miR-744 enhances IFN-induced CCL2, CCL5, CXCL10, and IL6 expression specifically in RMCs. We found that the activation of TYK2, STAT1 and STAT3 was significantly enhanced by miR-744. miR-744 also enhanced the activation of non-classical signal components, such as ERK and p38. We then identified PTP1B, a ubiquitously expressed phosphatase, as the target of miR-744 that is responsible for enhancing type I IFN response. Finally, miR-744 expression was induced by type I IFN in RMCs. Collectively, our data indicate that by targeting PTP1B, miR-744 plays a feed-forward role in regulating type I IFN signaling pathway. These findings give us new insights into the functions of renal miRNAs in regulating important signaling pathways.

Renal mesangial cells (RMCs) originate from monocytes or smooth muscle cells, and typically cover 30% of glomerular capillaries. As key glomerular cells, RMCs play an important role in the pathogenesis of several renal diseases^1^,^2^,^3^. Murine primary mesangial cells produce type I IFN when stimulated with poly I:C, which may be an important mediator of virus-induced glomerulonephritis^4^. Polyclonal anti-DNA antibodies from the sera of lupus patients bind significantly to the surfaces of RMCs^5^, leading to their activation. Activated RMCs generate many inflammatory molecules, including cytokines (interleukin 6 [IL6], IFN-γ, and IL12), chemokines (CCL2, CCL5, and CXCL10), and reactive oxygen mediators (reactive oxygen species and inducible nitric oxide synthase)^6^,^7^,^8^,^9^. Numerous inflammatory genes known to be upregulated in lupus nephritis^10^ are involved in mesangial abnormalities and the pathogenesis of lupus nephritis^11^,^12^,^13^,^14^. Therefore, it is very important to learn how these inflammatory cytokines and their related signaling pathways are regulated.

The significance of type I IFN in the predisposition to and amplification of autoimmunity and tissue damage^15^ indicated by many researches focusing on its activities increased our understanding of its function in normal and abnormal immune responses. Different genetic variants can lead to the overproduction of type I IFN in the peripheral blood mononuclear cells of patients with systemic lupus erythematosus (SLE). The increased bioavailability of type I IFN contributes to the over-activation of inflammatory factors which promote disease activity. Primary RMCs are capable of producing type I IFN and other inflammatory factors^4^ when stimulated with Toll-like receptor 3 (TLR3) ligands. Another study demonstrated that the PI:C RNA/TLR3-mediated disease activity of lupus nephritis depends on activated intrinsic renal cells, such as glomerular mesangial cells, which produce cytokines (such as IFN-α) and chemokines that aggravate autoimmune tissue injury^9^. The activation of the local type I IFN signaling pathway in kidney tissues was recently shown to be critical for the pathogenesis of lupus nephritis in a pristane-induced mouse model of lupus^16^.

miRNAs are emerging as important negative regulators of many kinds of biological processes^17^,^18^, acting via mRNA degradation or translational repression^19^. It has been proposed that miRNAs mediate important gene-regulatory events by targeting approximately 30% of the whole human transcriptome^20^. The contribution of miRNAs to the development and differentiation of immune cells has been gradually recognized. Several miRNAs play important roles in innate^21^,^22^ and adaptive immunity^23^,^24^,^25^. In recent years, scientists have discovered that miRNA levels correlate closely with SLE^26^,^27^,^28^ and other rheumatic diseases. A comprehensive analysis of miRNA expression in renal biopsies from patients with lupus nephritis showed that the expression of some miRNAs was abnormal^29^, possibly contributing to renal injury. However, the role of miRNAs in glomerular mesangial cells is not known.

Although several miRNAs have roles in regulating the type I IFN signaling pathway, the contribution of miRNAs to type I IFN signal transduction in primary human RMCs remains largely unclear. In this study, we investigated the involvement of miR-744 in primary human RMCs. miR-744 was first detected by Berezikov *et al.*^30^ and is highly conserved across all species. The literature on the functions of this miRNA is sparse; however, it was found to be significantly upregulated in the renal tissues of mice with diabetic nephropathy^31^, suggesting a role in kidney disease. Here, we identified a feed-forward role of miR-744 in the type I IFN pathway in human RMCs, but not in other types of cells that originate from different tissues. We demonstrate that miR-744 positively enhances the expression of IFN-induced genes (such as *CCL2*, *CCL5*, *CXCL10*, and *IL6*), and that the expression of these inflammatory genes is suppressed by the inhibition of miR-744. At the molecular level, miR-744 promotes the phosphorylation of both the canonical downstream signal components of the type I IFN pathway (such as TYK2, STAT1, and STAT3) and components of non-canonical pathways (such as p38 and ERK). Using an NF-κB inhibitor, we found that the non-canonical signaling pathway contributes most to enhancing the expression of those inflammatory genes. While exploring the potential mechanisms, we identified protein-tyrosine phosphatase 1B (PTP1B), a negative regulator of the insulin signaling pathway^32^, as the target of miR-744, which is responsible for the function of miR-744 in RMCs. Finally, we found that type I IFN induces miR-744 expression in RMCs. These findings suggest that miR-744 plays a vital feed-forward role in the type I IFN signaling pathway, specifically in human RMCs, providing important information for the further investigation of the roles of miR-744 in kidney diseases associated with an over-activated type I IFN signaling pathway (such as lupus nephritis).

## Results

### miR-744 enhances the expression of type-I-IFN-inducible genes in primary human RMCs

**Figure 1:**
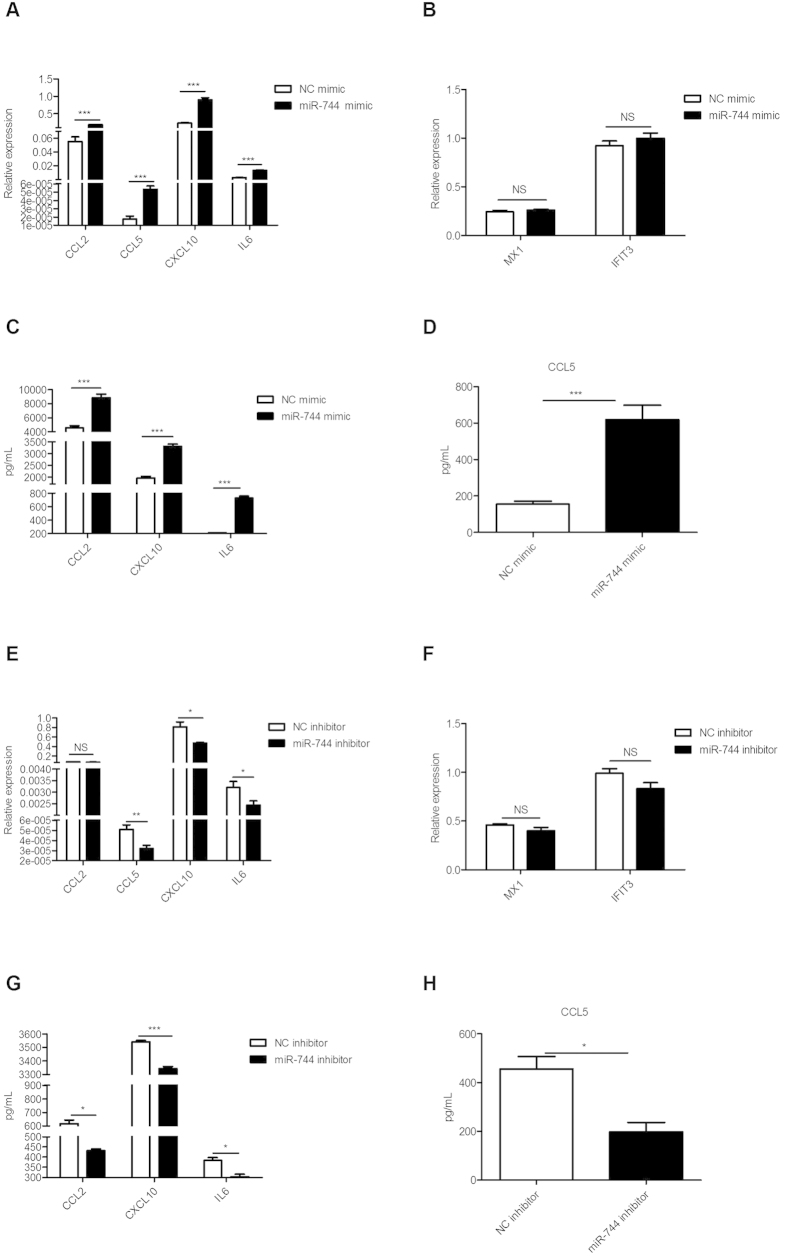
miR-744 enhances the expression of type-I-IFN-inducible genes in primary human RMCs. RMCs were transfected with an miR-744 mimic (200 nM) or negative control (NC) mimic (200 nM) 24 h before type I IFN was added. The cells were harvested after 6 h. The relative expression of type-I-IFN-inducible genes was detected with real-time PCR: *CCL2*, *CCL5*, *CXCL10*, and *IL6* are shown in (**A**); *MX1* and *IFIT3* are shown in (**B**). Supernatants harvested at 6 h were used to detect protein levels of CCL2, CXCL10, and IL6 (**C**); Supernatants harvested at 24 h were used to detect protein levels of CCL5 with an ELISA (**D**). RMCs were treated with an miR-744 inhibitor (400 nM) or control inhibitor (400 nM) for 48 h, then the cells were incubated for 6 hours with type I IFN. The relative expression of type-I-IFN-inducible genes was measured with real-time PCR: *CCL2*, *CCL5*, *CXCL10*, and *IL6* are shown in (**E**); *MX1* and *IFIT3* are shown in (**F**). Supernatants harvested at 6 h were used to measure protein levels of CCL2, CXCL10, and IL6 (**G**); Supernatants harvested at 24 h were used to measure protein levels of CCL5 with an ELISA (H).*p ≤ 0.05; **p ≤ 0.01; ***p ≤ 0.001; NS, not significant.

To examine the effects of miR-744 on the expression of type-I-IFN-inducible genes, we used mimics (synthesized RNA oligos the same as miR-744) to overexpress miR-744 and inhibitors (synthesized RNA oligos complementary to miR-744) to inhibit miR-744. We verified successful overexpression and inhibition of miR-744 by measuring the change of miR-744 expression (Supplementary Fig. S1A and Supplementary Fig. S1B). The overexpression of miR-744 promoted the expression of the inflammatory genes downstream of type I IFN, such as *CCL2*, *CCL5*, *CXCL10*, and *IL6*, at both the mRNA and protein levels, compared with cells transfected with the normal control (NC) (p < 0.01) (Fig. 1A,C,D). While the miR-744 inhibitor suppressed the expression of those IFN-inducible inflammatory genes (except *CCL2*) 6 h after stimulation with type I IFN (Fig. 1E). *CCL2* expression was also down-regulated by the miR-744 inhibitor 24 h after type I IFN stimulation (Supplementary Fig. S1C). Consistent with this, the inhibitory effects of the miR-744 inhibitor reduced the CCL2, CCL5, CXCL10, and IL6 protein levels (Fig. 1G,H). Intriguingly, miR-744 had no effect on the expression of traditional antiviral IFN-inducible genes, such as *MX1* and *IFIT3* (Fig. 1B,F). To determine whether the regulation of type I IFN signaling by miR-744 in human RMCs can be extended to other cells, we used two other cell lines originating from different tissues (Hela cells and HEK293T cells). In Hela and HEK293T cells, these genes were either not affected or reduced or promoted and the extent was not very big (Supplementary Fig. S2A, Supplementary Fig. S2B), which was not consistent with the effects caused by miR-744 in RMCs.

### miR-744 enhances both the classical JAK–STAT pathway and non-classical MAPKs and NF-κB pathways downstream of type I IFN

**Figure 2:**
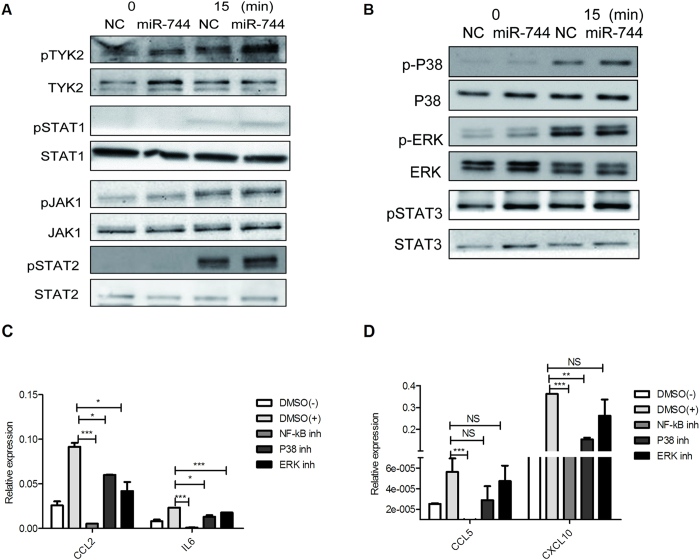
miR-744 enhances the classical JAK–STAT pathway and non-classical MAPK and NF-κB pathways downstream of type I IFN. Cells were transfected with miR-744 mimic or NC mimic (200 nM) for 24 h, then stimulated with type I IFN for 0 min or 15 min. Proteins were harvested and analyzed with western blotting to detect the phosphorylation of TYK2, STAT1, JAK1, and STAT2 (**A**) in the JAK–STAT pathway and p38, ERK, and STAT3 in the non-classical pathway (**B**) (The full-length blots/gels of TYK2, STAT1, JAK1, STAT2, p38, ERK and STAT3 are presented in Supplementary Fig. S4, Fig. S5, Fig. S6, Fig. S7, Fig. S8, Fig. S9, Fig. S10, respectively). RMCs were pretreated with SB203580 (p38 inhibitor), PD98059 (ERK inhibitor), or PDTC (NF-κB inhibitor) for 0.5 h before stimulation with type I IFN for 6 h. RNA was then harvested and the expression of IFN-inducible inflammatory genes was detected (**C,D**). *p ≤ 0.05; **p ≤ 0.01; ***p ≤ 0.001; NS, not significant.

To clarify the mechanism of miR-744 in regulating type I IFN signaling, we used western blotting to test whether the overexpression of miR-744 affected the JAK–STAT signaling pathway. The activation and phosphorylation of downstream signaling components of type I IFN signaling pathway, such as TYK2, STAT1, are very fast after the binding of IFN to its receptor^33^,^34^. So, we measured the phosphorylation of TYK2 and STAT1 by western blot. The results showed that overexpression of miR-744 promoted the phosphorylation of TYK2 and STAT1 (Fig. 2A). However, miR-744 had no apparent effect on the phosphorylation of JAK1 and STAT2 (Fig. 2A).

The expression of some IFN-inducible inflammatory genes (such as *CCL2*, *CCL5*, *CXCL10*, and *IL6*) was significantly up-regulated by miR-744 in human RMCs, whereas the expression of other IFN-inducible antiviral genes (such as *MX1* and *IFIT3*), which are mainly induced through the classical JAK–STAT signaling pathway^35^, were largely unaffected by miR-744. Therefore, we speculated that other signaling events other than the JAK–STAT signaling pathway were regulated by miR-744 in human RMCs.

Mitogen-activated protein kinases (MAPKs), such as ERK, p38, and NF-κB, which also participate in type I IFN signaling^36^,^37^, play important roles in inflammation by inducing the expression of inflammatory genes, including *CCL2*, *CCL5*, *CXCL10*, and *IL6*^38^,^39^. STAT3, which is highly expressed in human RMCs (data not shown), is also activated by type I IFN and is responsible for the expression of some chemokines^40^,^41^. Therefore, we tested whether miR-744 affects the type-I-IFN-induced phosphorylation of ERK, p38, and STAT3. The results showed that overexpressed miR-744 caused a considerable increase of the phosphorylation of p38 and STAT3 at 15 min (Fig. 2B). While, it showed modest increase of the phosphorylation of ERK at 15 min, but significant increase at 30min (Supplementary Fig. S9).

To confirm that MAPKs and NF-κB participate in regulating the expression of these inflammatory genes, we treated human RMCs with specific inhibitors of p38 (SB203580), ERK (PD98059), and NF-κB (PDTC) before adding type I IFN. The p38 inhibitor affected the expression of CCL2, IL6 (Fig. 2C), and CXCL10 (Fig. 2D), but not CCL5 (Fig. 2D); the ERK inhibitor affected the expression of CCL2 and IL6 (Fig. 2C), but not CXCL10 or CCL5 (Fig. 2D); and the NF-κB inhibitor had a marked inhibitory effect on the expression of all these genes in human RMCs (Fig. 2C,D).

Consistent with our previous data, miR-744 did not affect the downstream signaling events of type I IFN in another two cell types (Hela and HEK293T), and no changes were observed in the phosphorylation of STAT1 or STAT3 when miR-744 was overexpressed in Hela cells or HEK293T cells (Supplementary Fig. S2C, Supplementary Fig. S2D).

### miR-744 regulates the type I IFN signaling pathway by targeting PTP1B in human RMCs

**Figure 3:**
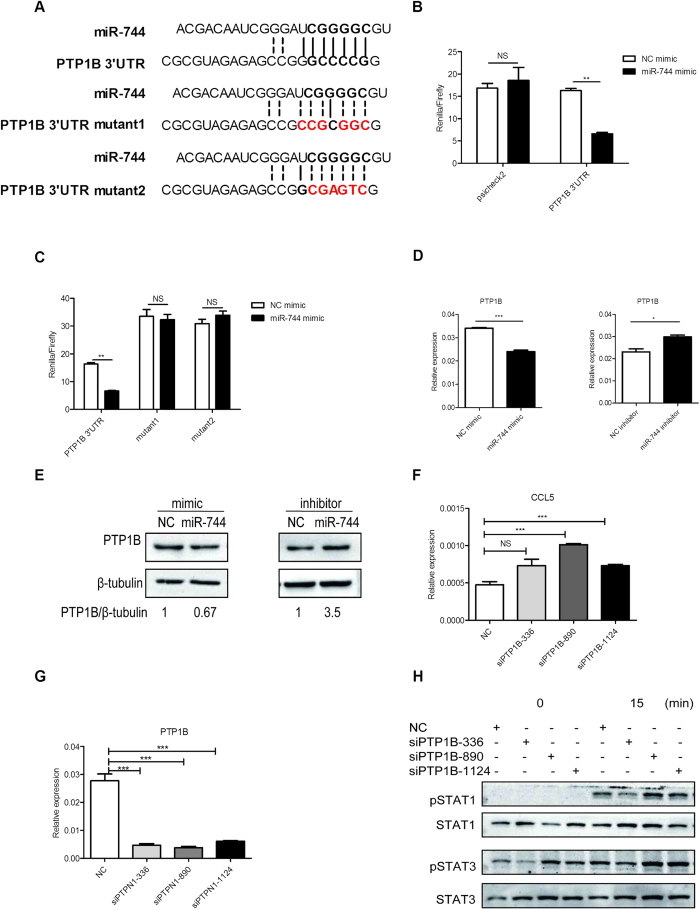
miR-744 targets *PTP1B*, which is responsible for its regulation of type I IFN signaling pathway. (**A**) Schematic diagram of potential miR-744 binding sites in the 3′-UTR of *PTP1B*, predicted by RNAhybrid, and two mutant binding sites. Mutant 1 abolished the binding to miR-744 without changing the nucleotide composition of the sequence, while mutant 2 affected both the nucleotide composition of the sequence and the binding of the 3′-UTR to miR-744. (**B**) RMCs were simultaneously transfected with NC or miR-744 (200 nM) and the *PTP1B* 3′-UTR-containing vector (50 ng per well) or pSicheck2 vector (50 ng per well). Luciferase activity was measured 24 h after transfection. (**C**) RMCs were simultaneously transfected with NC or miR-744 (200 nM) and the *PTP1B* 3′-UTR-containing vector (50 ng per well) or *PTP1B* 3′-UTR mutant vector (50 ng per well). Luciferase activity was measured 24 h after transfection, quantified, and expressed as relative luciferase activity. (**D**) RMCs were transfected with miR-744 mimic or inhibitor and the corresponding control mimic or inhibitor for either 24 h for the mimics (left) or for 48 h for the inhibitors (right). The levels of PTP1B mRNA were detected after stimulation with type I IFN for 6 h. (**E**) RMCs were transfected with miR-744 mimic or inhibitor and the corresponding control mimic or inhibitor for either 24 h for the mimics or for 48 h for the inhibitors. PTP1B was detected in the whole-cell lysates with western blotting. The ratios of PTP1B to β-tubulin in the control-transfected cells was arbitrarily set at 1(The full-length blots/gels of PTP1B are presented in Supplementary Fig. S11, Fig. S12, respectively). (**F**) Induction of CCL5 after PTP1B was silenced with siRNA (200 nM) in RMCs. (**G**) Efficiency of siRNA measured with real-time PCR. (**H**) Western blotting analysis of the phosphorylation of STAT1 and STAT3 in PTP1B-silenced RMCs. Cells were treated with type I IFN (1000 U/mL) for the indicated times (The full-length blots/gels of STAT1 and STAT3 are presented in Supplementary Fig. S13, Fig. S14, respectively). At least three independent experiments were performed. *p ≤ 0.05; **p ≤ 0.01; ***p ≤ 0.001. NS, not significant.

Our findings thus far have shown that miR-744 is a positive regulator of type I IFN signaling. To identify the molecular mechanisms of miR-744 functions, we used a bioinformatic tool to predict the potential targets of miR-744. We compiled a list of all the key negative regulators of the IFN signaling pathway and searched each of their genes for potential miR-744-binding sites with an algorithm called RNAhybrid (available at  http://bibiserv.techfak.uni-bielefeld.de/rnahybrid/submission.html). We found that miR-744 base-paired with sequences in the 3′-untranslated region (UTR) of *PTP1B* (Fig. 3A). PTP1B is a ubiquitously expressed phosphatase that has been implicated in various signaling pathways^42^. Studies have shown that TYK2 and JAK2 are physiological substrates of PTP1B, which dephosphorylates the tyrosine phosphorylation of these two kinases^43^. PTP1B also dephosphorylates STAT3, p38, and ERK and suppresses NF-κB–p65-mediated transcription^44^,^45^,^46^,^47^. Therefore, *PTP1B* may be a promising target of miR-744.

To confirm the targeting relationship between miR-744 and *PTP1B*, we conducted a biological validation. First, the 3′-UTR fragment of *PTP1B* was cloned downstream of a luciferase reporter gene. Human RMCs were then transiently transfected with the construct, together with either an miR-744 mimic or an NC mimic. As expected, the overexpression of miR-744 effectively attenuated the luciferase activity, compared with NC (Fig. 3B). To confirm that miR-744 binds to the 3′-UTR of *PTP1B*, we constructed two target-site mutant vectors (Fig. 3A) and transfected RMCs with them, as described above. As expected, mutation of the 3′-UTR of *PTP1B* abolished the inhibitory effect of miR-744 (Fig. 3C), implying that the inhibitory effect of miR-744 requires the predicted binding sites. Then, we found that overexpressing miR-744 in RMCs reduced the mRNA levels of PTP1B (Fig. 3D, left), while inhibition of miR-744 increased the mRNA levels of PTP1B (Fig. 3D, right). And consistently, we found that overexpressing miR-744 in RMCs reduced the protein levels of PTP1B by western blot (Fig. 3E, left), while inhibition of miR-744 increased the protein levels of PTP1B (Fig. 3E, right). We analyzed the band signal intensities of western blot results and calculated the relative protein expression levels of PTP1B. And by statistical analysis we found that PTP1B was indeed reduced by the overexpression of miR-744 and up-regulated by the inhibition of miR-744 inhibitors in RMCs (Supplementary Fig. S3A, Supplementary Fig. S3B). We knocked down the expression of PTP1B in RMCs using a small interfering RNA (siRNA) (Fig. 3G and Supplementary Fig. S3C show the efficiency of the siRNA). Our data showed that silencing PTP1B promoted type I IFN signaling in RMCs, with elevated levels of CCL5 (Fig. 3F), and enhanced the phosphorylation of STAT1 and STAT3 (Fig. 3H), mimicking the effects of miR-744 overexpression. Therefore, we concluded that miR-744 positively regulates the type I IFN signaling pathway in human RMCs by targeting *PTP1B*.

### Type I IFN increases the expression of miR-744 in human RMCs

**Figure 4:**
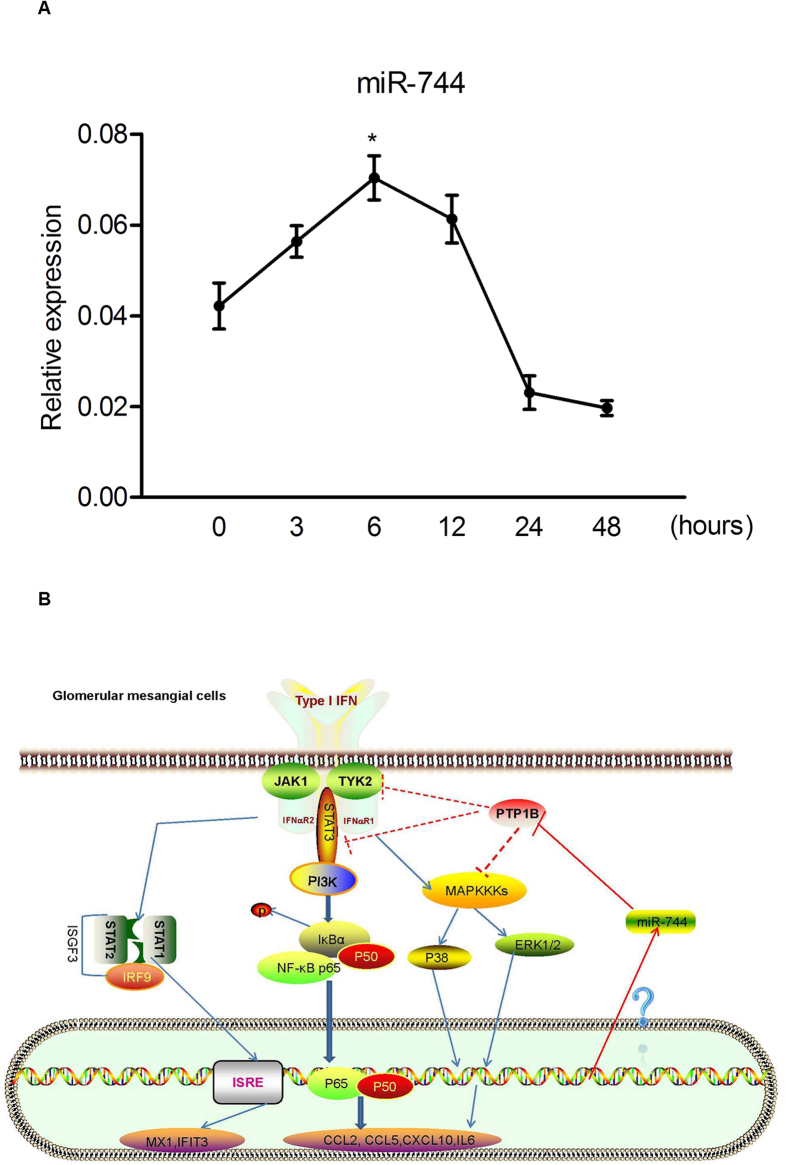
miR-744 is induced by type I IFN and may act as a feed-forward regulator of the type I IFN signaling pathway. (**A**) RMCs were stimulated with type I IFN (1000 U/mL) and the RNA was collected at the indicated time points. The expression of miR-744 was detected with qRT–PCR. Each point shows the mean relative expression level of miR-744 for three independent experiments. We used *RNU48* as the reference gene. (**B**) Schematic diagram of the mechanisms by which miR-744 feed-forward regulates the IFN-activated classical JAK–STAT and non-classical MAPK and NF-κB signaling pathways. Type I IFN triggers an unknown transcription factor to induce miR-744 expression. miR-744 subsequently represses PTP1B expression, leading to the enhanced activation of TYK2, STAT1, STAT3, ERK, p38, and NF-κB, thus enhancing the expression of IFN-induced inflammatory genes.

The results described above demonstrate that miR-744 positively regulates the type I IFN signaling pathway in RMCs by targeting PTP1B in the signaling cascade. We were interested to know whether miR-744 levels are affected by type I IFN. Therefore, we tested the expression of miR-744 in RMCs stimulated with type I IFN for specific times. We observed the expression of miR-744 increased at 3 h and peaked at 6 h, after which it gradually declined (Fig. 4A). These results indicate that miR-744 is induced by type I IFN. Based on the data described above, miR-744 might act as a feed-forward regulator of the type I IFN signaling pathway (Fig. 4B).

## Discussion

Previous studies have shown that RMCs from patients with lupus nephritis or diabetic nephropathy display numerous signaling abnormalities, which contribute to the occurrence of the disease. Activated RMCs produce chemokines and cytokines, such as CCL2, CCL5, CXCL10, and IL6^38^,^39^,^48^. These inflammatory proteins contribute directly to mesangial proliferation or recruit immune cells to the renal mesangium, causing intense inflammation^49^, which plays a vital role in glomerular diseases, especially lupus nephritis. Therefore, it is important to determine the regulatory mechanisms of important signaling pathways in RMCs.

Ever since the discovery of miRNAs, growing evidence has demonstrated that miRNAs are associated with the progression and prognosis of several diseases, and may be potential drug targets. Abundant evidence has indicated that miRNAs regulate signaling events, and defects in their expression cause abnormalities in cytokine signaling pathways. For example, the expression of miR-146a correlates negatively with SLE disease activity and IFN scores^50^, and miR-125a elevates RANTES levels by targeting KLF13 in T cells^51^.

Over-activated type I IFN signaling is critical to the pathogenesis of lupus nephritis^52^. Therefore, in this study, we selected primary human RMCs and the type I IFN signaling pathway to explore the functions of mesangial miR-744.

The expression of miR-744 is significantly increased in the glomeruli of diabetic mice^31^, and miR-744 regulates transforming growth factor β signal transduction^53^. In this study, miR-744 clearly increased the expression of some type-I-IFN-inducible genes, such as *CCL2*, *CCL5*, *CXCL10*, and *IL6*, in human RMCs. However, the expression of genes associated with antiviral activities, such as *MX1* and *IFIT3*, did not change. Once induced, type I IFN binds to its receptors to initiate downstream signaling by activating the JAK–STAT pathway or other non-classical pathways, including the NF-κB^36^ and MAPKs pathways^37^, ultimately leading to the transcription of its target genes (Fig. 4B). The overexpression of miR-744 not only enhanced the phosphorylation of TYK2, STAT1, and STAT3, but also enhanced the activation of ERK and p38 by IFN. Further experiments demonstrated that the genes regulated by miR-744 depend most strongly on the activation of MAPKs and NF-κB. STAT3 is highly expressed in human RMCs (data not shown) and other studies have confirmed that type-I-IFN-activated STAT3 inhibits STAT1-dependent gene activation instead of inhibiting STAT1 activation in myeloid cells^54^. Collectively, the information described above at least partly explains why those antiviral genes, the transcription of which is activated by the STAT1-dependent transcription factor complex (ISGF3) (Fig. 4B), are not significantly affected by miR-744. Thus, miR-744 selectively regulates a subset of IFN-inducible genes in RMCs.

Cells spontaneously use various mechanisms to negatively regulate the type I IFN signaling pathway to avoid abnormal activation and to maintain an immunological balance in the physiological context. Defects in these negative regulators could lead to the over-activation of positive signal transduction, resulting in disease, such as SOCS1, which negatively regulates the lipopolysaccharide response^55^, and USP18, down-regulates the IFN signaling pathway^56^. We have shown that miR-744 targets *PTP1B* by binding to its 3′-UTR, thus dephosphorylating TYK2, STAT3, and MAPKs^43^,^45^,^46^, all of which are important mediators of both the classical and non-classical IFN signaling pathways. Therefore, miR-744 promotes the type I IFN signaling pathway (Fig. 4B).

Finally, to determine whether miR-744 is regulated by type I IFN, we treated RMCs with IFN and found that mature miR-744 is induced by type I IFN. Therefore, we propose here that miR-744 acts as a feed-forward component of the type I IFN signaling pathway (Fig. 4B).

The type I IFN signaling pathway has emerged as a significant contributor to the pathogenesis of lupus nephritis, in which RMCs are critical. Therefore, understanding the regulation of the IFN signaling pathway will allow the development of promising strategies for the treatment of this disease. However, these strategies should be applied with considerable caution because life-threatening immunodeficiencies can potentially be induced^57^. Because miRNAs quantitatively regulate gene expression rather than acting as on/off signals, they are ideal target molecules with which to fine-tune the cell’s responses to external signals^58^. Interestingly, when an miR-744 inhibitor was introduced into RMCs, the coordinated activation of the type I IFN signaling pathway was significantly reduced, as revealed by the down-regulated expression of several IFN-inducible genes. Because miR-744 does not affect genes with antiviral activities and its functions are cell-type specific, our results suggest that miR-744 has the potential to be one of the drug targets. It may be manipulated effectively to provide useful therapeutic interventions for diseases associated with abnormal IFN activities in RMCs, without inhibiting the antiviral activities of IFN, which still need more studies to verify.

In summary, our data show that miR-744 acts as a feed-forward component of the type I IFN signaling pathway in RMCs, and exclusively affects the expression of inflammatory chemokines and cytokines but not the expression of antiviral genes, by targeting *PTP1B*. These data extend our understanding of the regulation of the type I IFN signaling pathway in primary human RMCs. Further studies of renal biopsies from patients with lupus nephritis will show whether the expression of miR-744 is abnormal, to advance our knowledge of the pathogenesis of lupus nephritis and identify new drug targets.

## Materials and Methods

### Cells and reagents

Primary human RMCs (obtained from ScienCell Research Laboratory, Shanghai, China) were maintained in Mesangial Cell Medium (ScienCell) supplemented with 10% fetal bovine serum (FBS, ScienCell), 100 U/mL penicillin, and 100 μg/mL streptomycin (ScienCell) at 37 °C in a humidified atmosphere containing 5% CO_2_. Hela cells (obtained from Cell Bank, Shanghai Institutes for Biological Sciences, Chinese Academy of Sciences, Shanghai, China) and HEK293T cells (obtained from Cell Bank) were grown in Dulbecco’s modified Eagle’s medium (Gibco) supplemented with 10% FBS (ScienCell) at 37 °C in a humidified atmosphere containing 5% CO_2_.

Primary human RMCs from ScienCell Research Laboratory were collected from donors, with the signed informed consent of the donor themselves or of an authorized agent acting on the donor’s behalf.

The miRNA mimics and inhibitors were synthesized by Shanghai Genepharma Co., Ltd. PTP1B siRNA and a control siRNA were from Shanghai Genepharma Co., Ltd. The human *PTP1B* siRNA sequences were: PTP1B-homo-336 sense 5′-GUCGGAUUAAACUACAUCATT-3′, antisense 5′-UGAUGUAGUUUAAUCCGACTT-3′; PTP1B-homo-890 sense 5′-GACCCUUCUUCCGUUGAUATT-3′, antisense 5′-UAUCAACGGAAGAAGGGUCTT-3′; and PTP1B-homo-1124 sense 5′-GAGCCACACAAUGGGAAAUTT-3′, antisense 5′-AUUUCCCAUUGUGUGGCUCTT-3′.

For pathway screening, RMCs were stimulated with type I IFN (1,000 units/mL; PBL Interferon Source, Piscataway, NJ) alone or in the presence of various inhibitors, including 50 μM pyrrolidine dithiocarbamate (Tocris), 10 μM PD98059 (Tocris), or 5 μM SB203580 (Tocris).

### Transfection

Cells were transfected with miRNA mimics, miRNA inhibitors, or siRNA using Lipofectamine® RNAiMAX Transfection Reagent (Invitrogen), according to the manufacturer’s instructions. Transfection was typically performed in cells that were 80% confluent. To analyze the expression of inflammatory genes, cells were seeded in 24-well plates at a concentration of 5 × 10^4^ per well in 500 μL of medium.

### Analysis of mRNA and miRNA with quantitative RT–PCR

RNA was extracted with TRIzol Reagent (Invitrogen), according to the manufacturer’s instructions. RNA quality was confirmed with spectrophotometers and approximately 200 ng was reverse transcribed into complementary DNA (cDNA) with the PrimeScript™ RT Reagent Kit (Takara). To quantify mRNA expression, the cDNA was amplified with real-time PCR using SYBR® Premix Ex Taq™ (Takara). The expression of *RPL13A* was used as the internal control.

miRNA was quantified by reverse transcribing 100 ng of RNA from each sample and detecting the miRNA with specific TaqMan probes using a TaqMan Human MicroRNA Assay Kit (Applied Biosystems). *RNU48* was used as the internal control.

The TaqMan and SYBR Green assays were performed in duplicate or triplicate on a ViiA7 Real-Time PCR System (Applied Biosystems). The relative expression levels were calculated with the 2^-ΔCt^ method.

### ELISAs

CCL2, CCL5, and IL6 proteins in the culture supernatants were measured with ELISA kits from R&D Systems. CXCL10 was detected with an ELISA kit from Shanghai Westang Bio-Tech Co., Ltd. The experiments were performed according to the manufacturers’ instructions.

### Western blotting

Cells were seeded in six-well plates at a concentration of 5 × 10^5^ per well in 1 mL of medium and transfected with miRNA mimics or miRNA inhibitors. The cells were stimulated with type I IFN (1000 U/mL) 24 h after transfection and the cells were lysed at the indicated time points. The proteins were extracted, separated with sodium dodecyl sulfate-polyacrylamide gel electrophoresis, blotted, and probed with the specified primary antibodies directed against phosphorylated STAT1 (p-STAT1), STAT1, p-STAT2, STAT2, JAK1, PTP1B, p-P44/42 MAPK (ERK1/2), P44/42 MAPK (ERK1/2), p38 MAPK, p-p38 MAPK, and p-TYK2 (Cell Signaling Technology, diluted 1:1000); anti-STAT3, anti-p-STAT3, and anti-p-JAK1 (Santa Cruz Biotechnology, diluted 1:200); anti-TYK2 and anti-β-tubulin (Abcam, diluted 1:1000 and 1:5000, respectively). The secondary antibodies used were obtained from Cell Signaling: horseradish-peroxidase (HRP)-linked anti-rabbit IgG antibody (diluted 1:5000), and HRP-linked anti-mouse IgG antibody (diluted 1:4000). The signal was generated with SuperSignal® West Pico Luminol/Enhancer Solution (Pierce). We used Tanon 6600 Luminescent Imaging Workstation (Tanon Science & Technology, Shanghai, China) to scan the film. And band signal intensity was analyzed using Image J.

### Preparation of constructs

To create 3′-UTR–luciferase reporter constructs, fragments of the 3′-UTR from the protein-tyrosine phosphatase 1B (*PTP1B*) gene, containing the predicted miR-744-binding site, were cloned downstream of the firefly luciferase gene in the psiCHECK™-2 vector (Promega). The *PTP1B* 3′UTR was PCR amplified with the following primers: forward 5′-GAATCCGCTCGAGACATAGCCTGACCCTCCTC-3′ and reverse 5′-TAATAGCGGCCGCCTTACAACCGTCCTCCTTC-3′. Mutations were introduced with KOD FX DNA polymerase (Toyobo) and the following primers: mutant1, forward 5′-GTAGAGAGCCGCCGCGGCGACGGACGTTGG-3′, reverse 5′-CCAACGTCCGTCGCCGCGGCGGCTCTCTAC-3′; mutant2, forward 5′-CGTAGAGAGCCGGCGAGTCGACGGACGTT-3′, reverse 5′-AACGTCCGTCGACTCGCCGGCTCTCTACG-3′. All constructs were verified with DNA sequencing.

### Luciferase reporter assay

Human RMCs were seeded in a 96-well plate at a concentration of 2 × 10^4^ per well in 100 μL of medium and transfected with a mixture of 50 ng of 3′-UTR luciferase reporter vector or empty vector and 200 nM miR-744 mimic or NC mimic with Lipofectamine 2000 (Gibco), according to the manufacturer’s instructions. The cells were lysed 24 h after transfection and their luciferase activity was measured with the Dual-Luciferase Reporter Assay System (Promega) and a luminometer (Berthold Technologies). The ratio of *Renilla* luciferase activity to *firefly* luciferase activity was calculated.

### Statistical analysis

The GraphPad Prism 5 statistical software was used for all statistical analyses. Data were expressed as the means ± SEM of at least three independent experiments. The significance of differences was measured with Student’s *t* test for comparisons of two groups or with analysis of variance (ANOVA) followed by Turkey’s *t* test for comparisons of multiple groups. And p < 0.05 was considered statistically significant.

## Additional Information

**How to cite this article**: Zhang, X. *et al.* miR-744 enhances type I interferon signaling pathway by targeting PTP1B in human primary renal mesangial cells. *Sci. Rep.* **5**, 12987; doi: 10.1038/srep12987 (2015).

## Supplementary Material

Supplementary Information
