# Supplementary material for: miR-744 enhances type I interferon signaling pathway by targeting PTP1B in primary human renal mesangial cells
Source: Sci Rep. 2015 Aug 11;5:12987. doi: 10.1038/srep12987 (PMC4531339; doi:10.1038/srep12987)
Supplement: Supplementary Information [file srep12987-s1.pdf]

# miR-744 enhances type I interferon signaling pathway by targeting PTP1B in primary human renal mesangial cells

**Xiaoyan Zhang[1#], Xiao Han[2#], Yuanjia Tang[1], Yanfang Wu[1], Bo Qu[1*], Nan Shen[1, 2, 3*]**

[1]Shanghai Institute of Rheumatology, Department of Rheumatology, Ren Ji Hospital, School of Medicine, Shanghai Jiao Tong University, Shanghai, China

[2] Institute of Health Sciences, Shanghai Institutes for Biological Sciences (SIBS) & Shanghai Jiao Tong University School of Medicine (SJTUSM), Chinese Academy of Sciences (CAS), Shanghai, China

[3]Division of Rheumatology and the Center for Autoimmune Genomics and Etiology (CAGE), Cincinnati Children's Hospital Medical Center, Cincinnati, Ohio, United States of America

***Correspondence to**: Nan Shen, Email: nanshensibs@gmail.com.

Or to: Bo Qu, Email: qubo.bio@gmail.com.

[#] These authors contributed equally to this work.

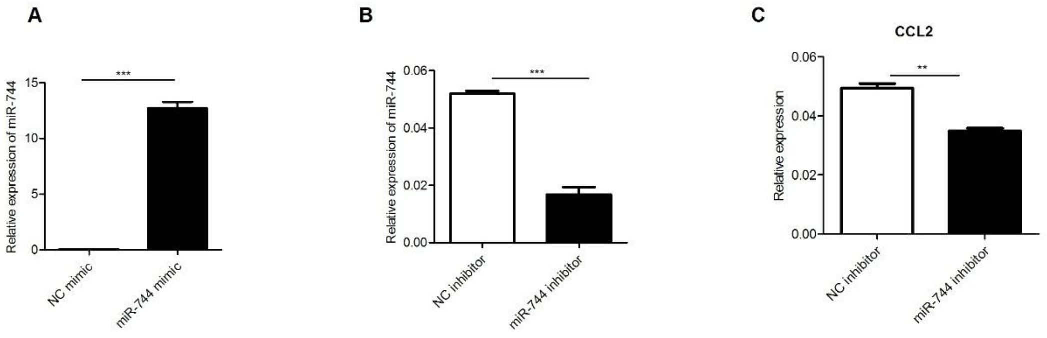


**Figure S1. The efficiency of overexpression and silencing miR-744 and the effect of miR-744 inhibitors on CCL2 gene expression.** We transfected RMCs with miR-744 mimics or NC mimics at 200 nM (A) and miR-744 inhibitors or NC inhibitors at 400 nM (B). Then qPCR were used to determine the expression of miR-744. (C) RMCs were transfected with miR-744 inhibitors or NC inhibitors (400 nM) for 48 h, then cells were stimulated with type I IFN for 24 h. Then CCL2 was measured by qPCR.

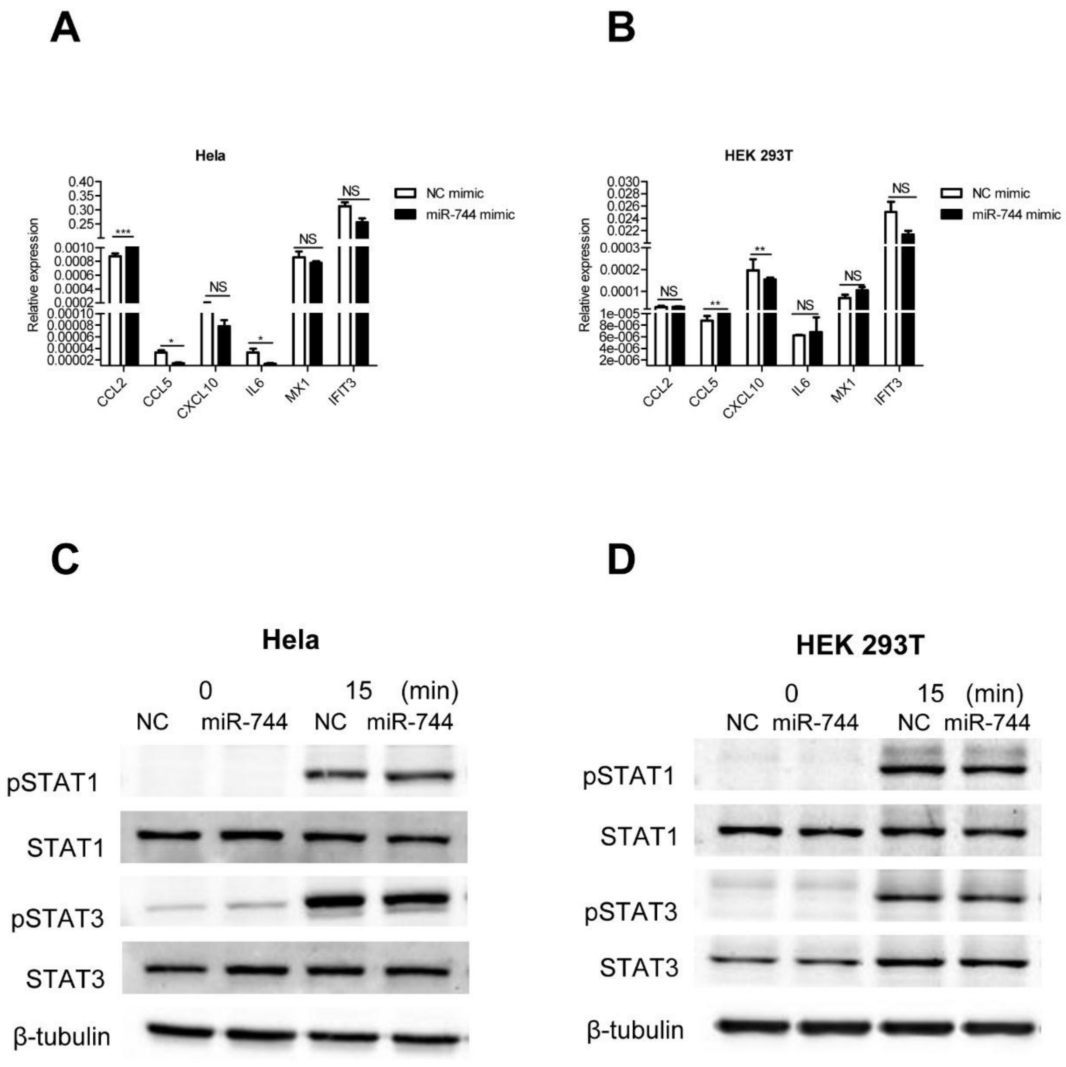


**Figure S2. The effects of miR-744 on type I IFN signaling pathway in Hela and HEK 293T cells.** Cells were transfected with miR-744 mimics or NC mimics (100 nM). 24 h later, cells were stimulated with type I IFN for 6 h. Inflammatory genes were detected by qPCR in Hela cells (A) and HEK 293T cells (B), respectively. Cells were incubated with type I IFN for 15 min, then protein were harvested. And the phosphorylation of STAT1 and STAT3 were detected by western blot in Hela cells(C) and HEK 293T cells (D) at the indicated time point.

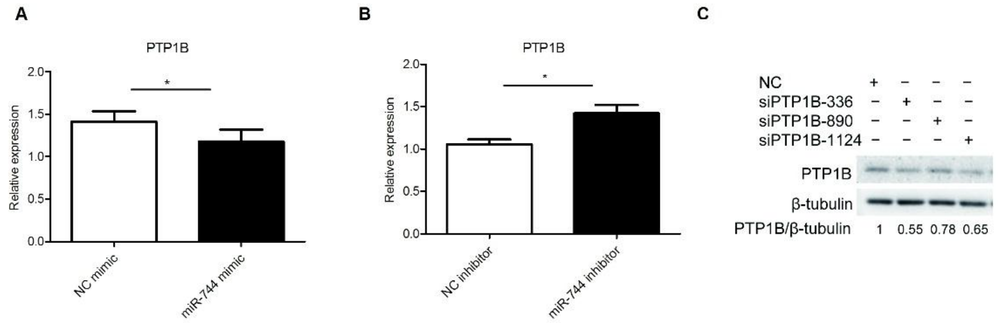


**Figure S3. Statistical analysis of the effects of miR-744 on the protein levels of PTP1B.** RMCs were transfected with either miR-744 mimics (A) or inhibitors (B) and their corresponding negative control (NC) mimics or inhibitors. Then protein were harvested and western blot was performed. The quantification of the intensity of band signal was done by Image J, the relative expression levels of PTP1B were normalized to β-tubulin (A and B). (C) The efficiency of PTP1B siRNA. RMCs were transfected with siRNAs or NC (200nM) for 24 h. Then protein were harvested and western blot was performed. The ratios of PTP1B to β-tubulin were calculated and the ratio of NC group were arbitrarily set as 1.

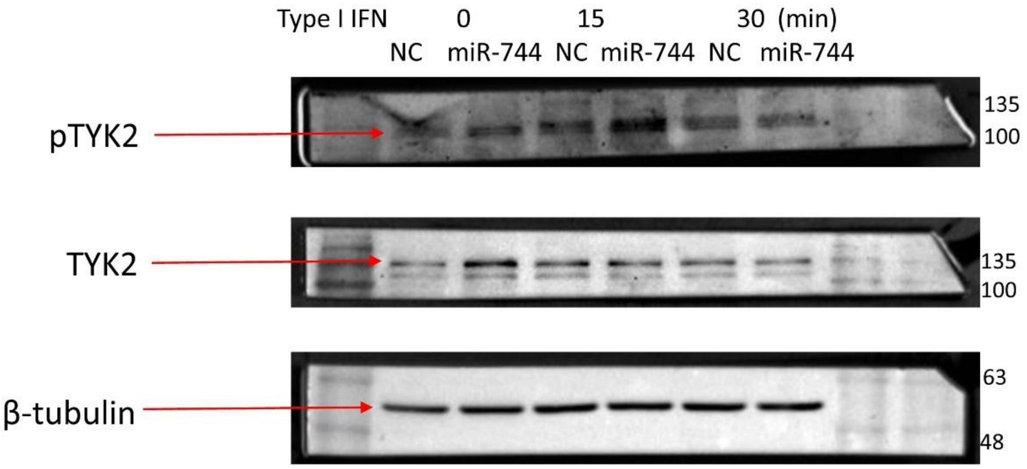


Figure S4：Full-length blots/gels of pTYK2 and TYK2 in the Figure 2A in the main paper.

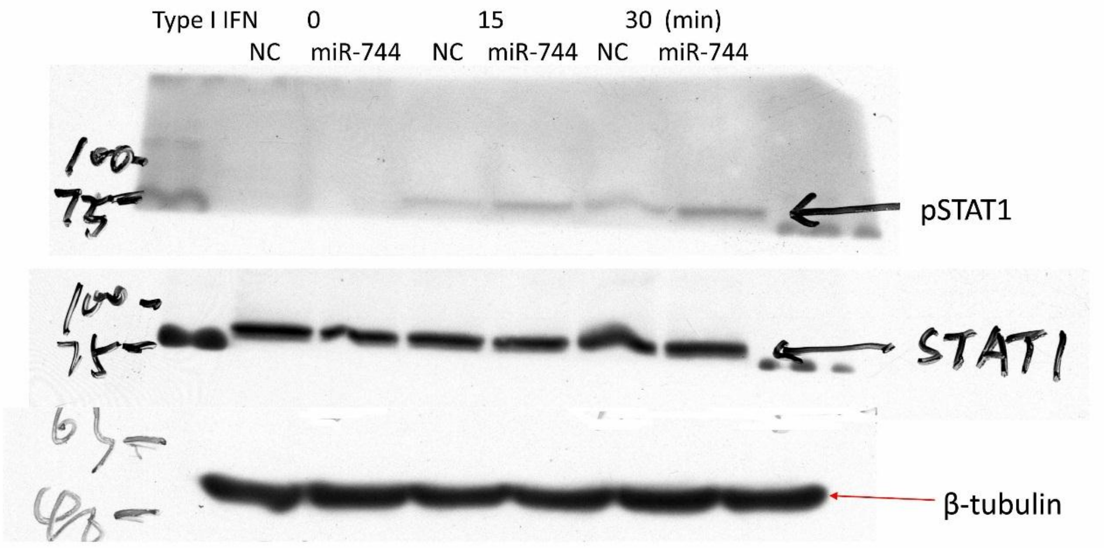


Figure S5：Full-length blots/gels of pSTAT1 and STAT1 in the Figure 2A in the main paper.

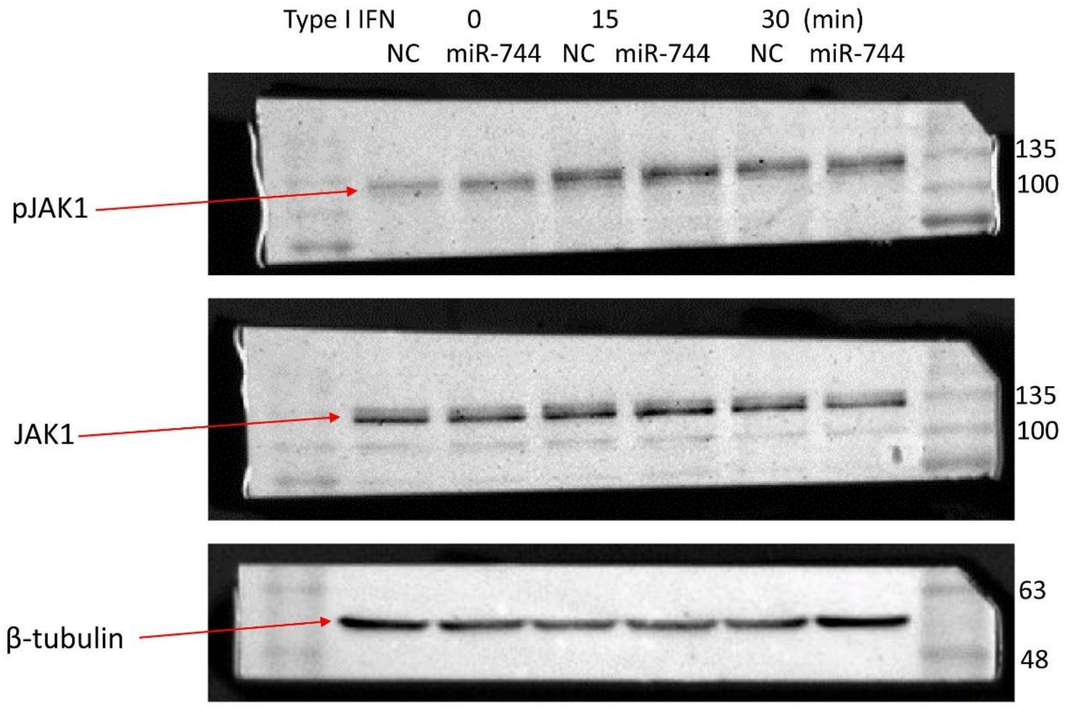


Figure S6：Full-length blots/gels of pJAK1 and JAK1 in the Figure 2A in the main paper.

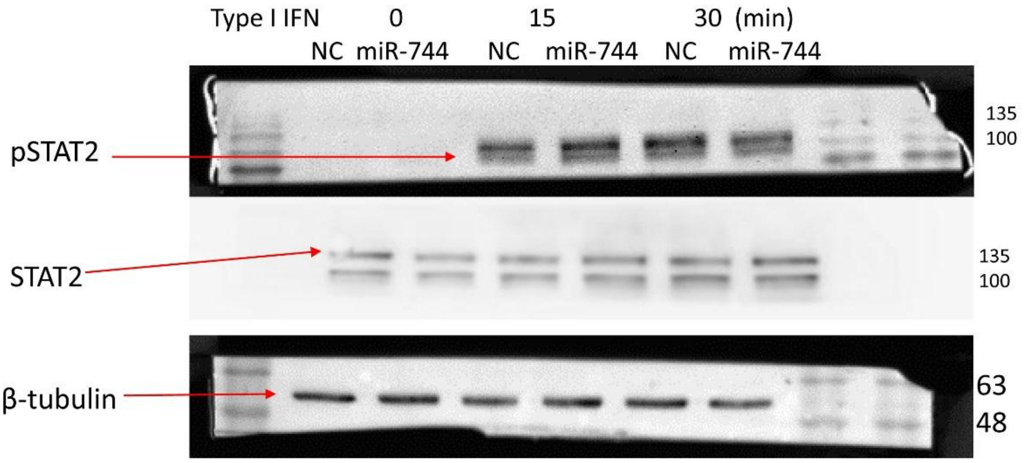


Figure S7：Full-length blots/gels of pSTAT2 and STAT2 in the Figure 2A in the main paper.

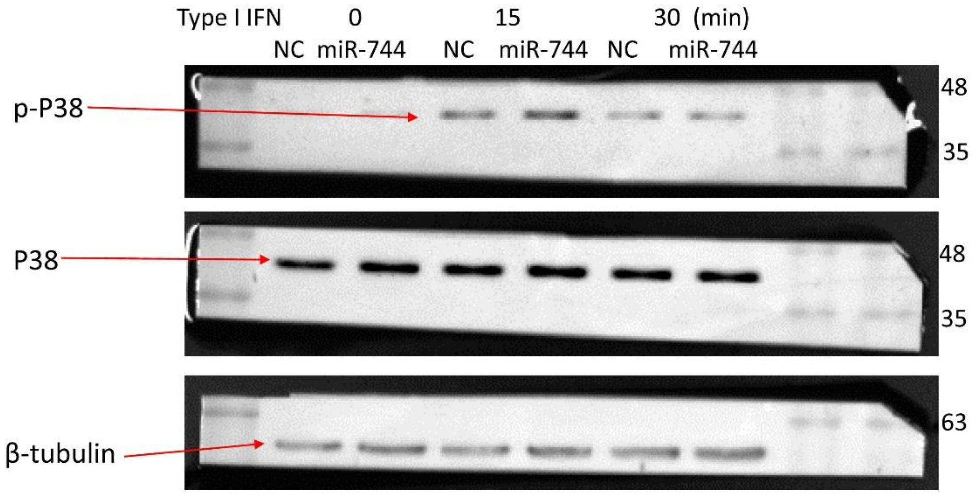


Figure S8：Full-length blots/gels of p-P38 and P38 in the Figure 2B in the main paper.

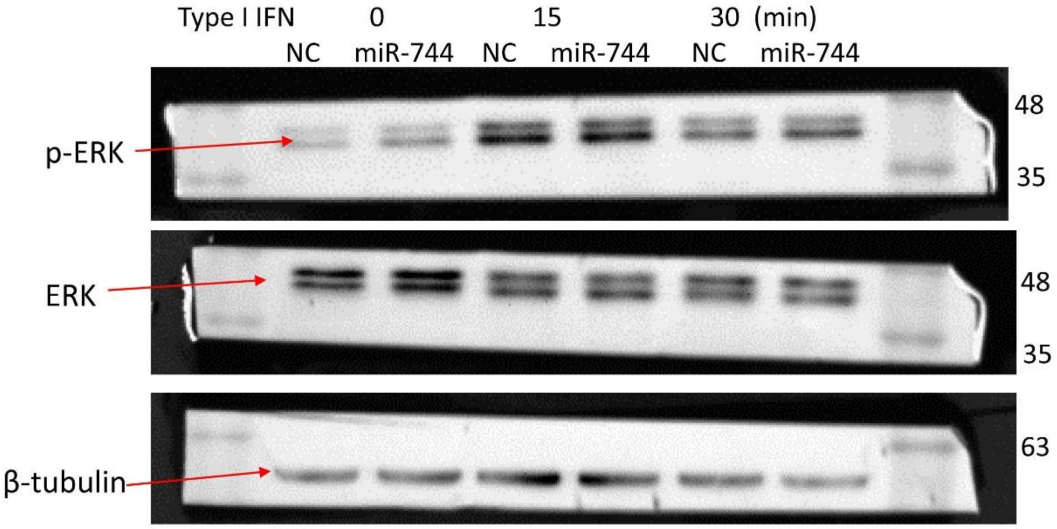


Figure S9：Full-length blots/gels of p-ERK and ERK in the Figure 2B in the main paper.

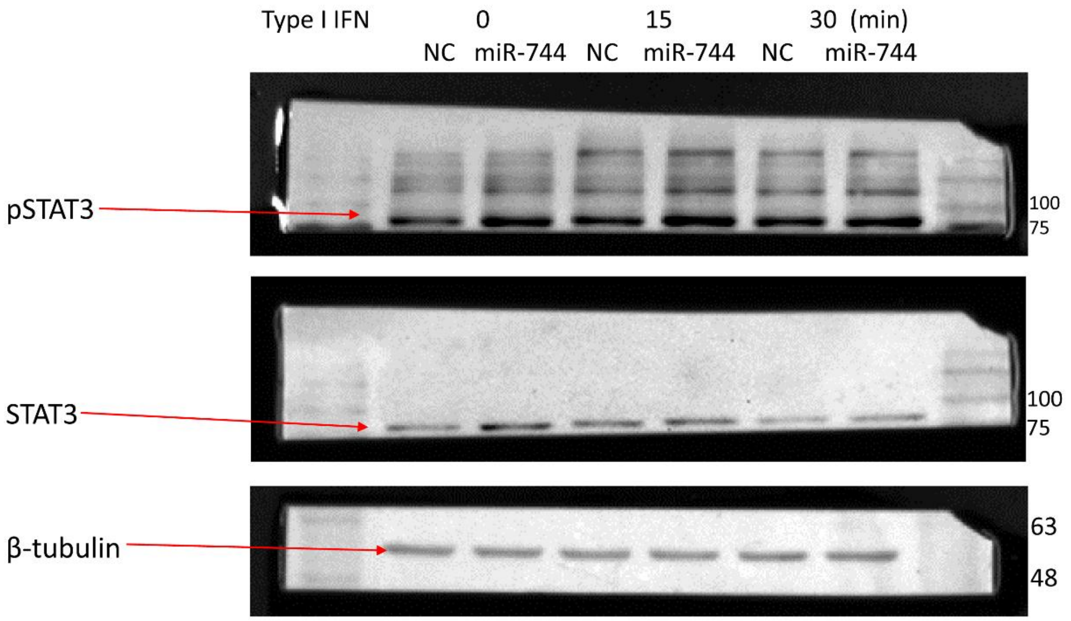


Figure S10：Full-length blots/gels of pSTAT3 and STAT3 in the Figure 2B in the main paper.

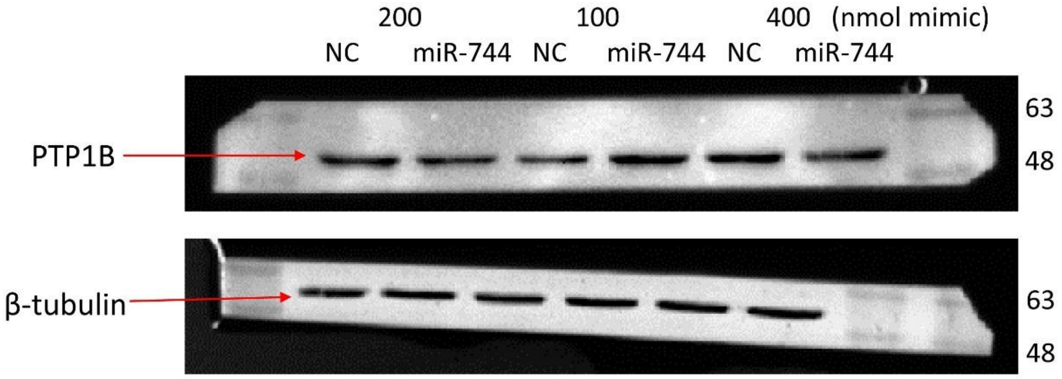


Figure S11：Full-length blots/gels of PTP1B in the Figure 3E (left) in the main paper.

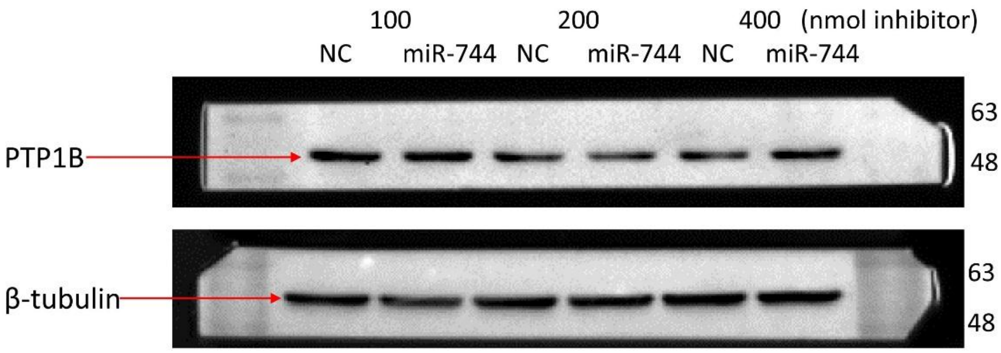


Figure S12：Full-length blots/gels of PTP1B in the Figure 3E (right) in the main paper.

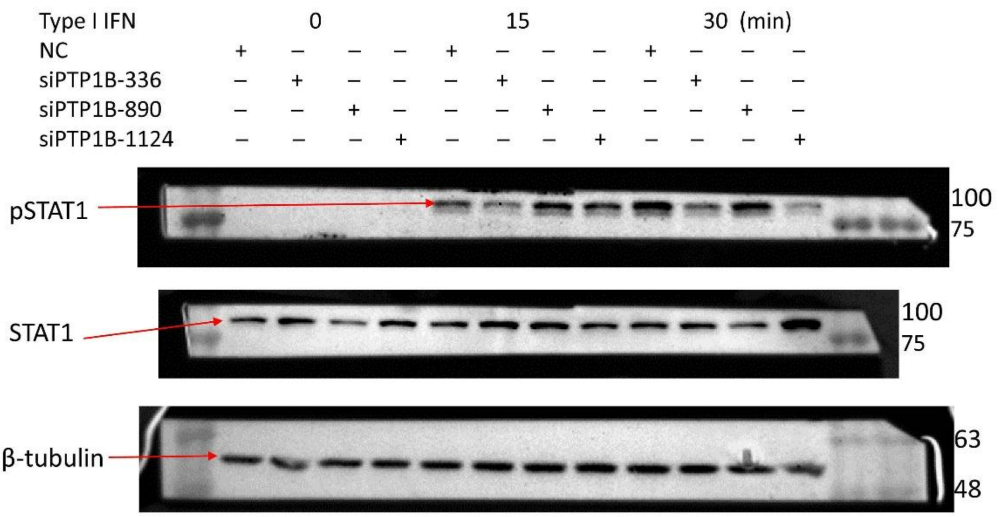


Figure S13：Full-length blots/gels of pSTAT1 and STAT1 in the Figure 3H in the main paper.

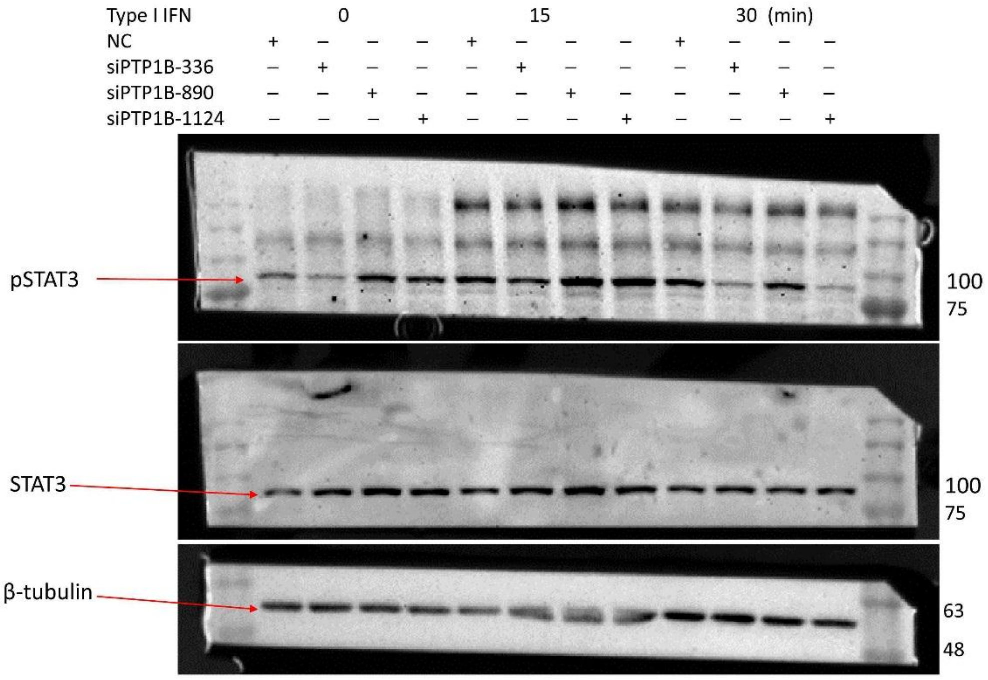


Figure S14：Full-length blots/gels of pSTAT3 and STAT3 in the Figure 3H in the main paper.
